# Supplementary figures and images for: Immunogenicity of SARS-CoV-2 Trimeric Spike Protein Associated to Poly(I:C) Plus Alum
Source: Front Immunol. 2022 Jun 30;13:884760. doi: 10.3389/fimmu.2022.884760 (PMC9281395; doi:10.3389/fimmu.2022.884760)

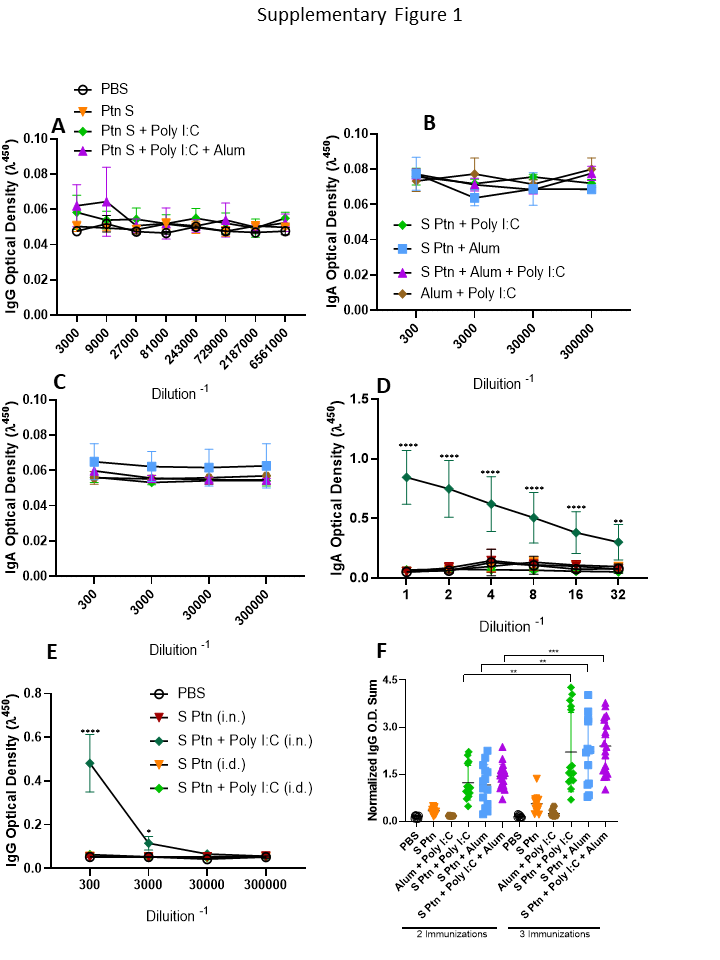

Supplement: Supplementary Figure 1 — The prime was not a good inducer of IgG, and IgA levels were very low after immunizations. Antigen-specific antibody levels were determined by ELISA and normalized using the control groups. Serum IgG levels (A) was evaluated 1 week after prime. Serum IgA levels (B, C) were evaluated after two (B) or three immunizations (C). BALF IgA (D) and IgG (E) levels. Data in (A–C) are representative of 4 independent experiments and are shown as mean ± S.D. Data in (D, E) are from a single experiment and are shown as mean ± S.D. Groups: PBS (n=5); S Ptn (n=5); Alum + Poly(I:C) (n=6); S Ptn + Poly(I:C) (n=5); S Ptn + Alum (n=5); S Ptn + Poly(I:C) + Alum (n=5). * - represents differences between S Ptn + Poly(I:C) (intradermal) and S Ptn + Poly(I:C) (intranasal) groups (D, E) and analyzed by two-way ANOVA followed by Bonferroni post-test and (F) was analyzed by one-way ANOVA with Tukey’s post hoc test. *p<0.05, **p<0.01, ***p<0.001, ****p<0.0001. [file Image_1.tif]

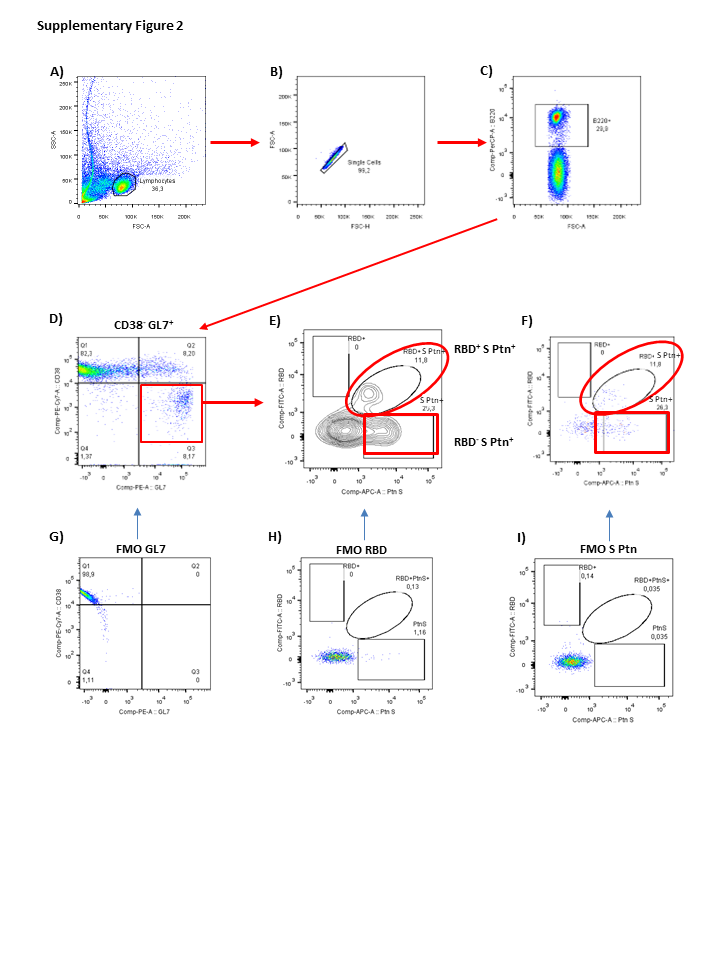

Supplement: Supplementary Figure 2 — Gating strategy of B cells in draining lymph nodes. Lymphocytes from the draining popliteal lymph node were analyzed after three immunizations as follows: (A) Cells FSC x SSC. (B) Single cells - FSC-A x FSC-H. (C) B220+ (PerCP x FSC-A). (D) CD38-GL7+ (PE-Cy7 x PE). (E, F) RBD+S Ptn+ (FITC x APC). (G) FMO GL7. (H) FMO RBD. (I) FMO S Ptn. [file Image_2.tif]

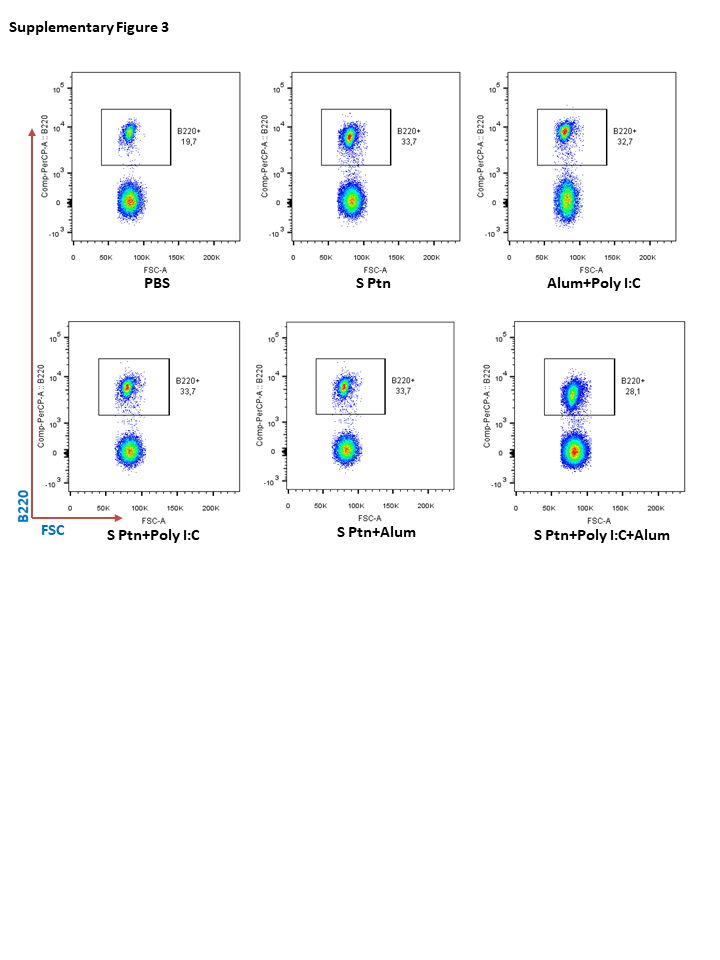

Supplement: Supplementary Figure 3 — Dot plot of B220+ cells. Lymphocytes isolated from the draining popliteal lymph node after three immunizations by intradermal immunization with S Ptn alone or with adjuvants (Poly(I:C); Alum; Poly(I:C) + Alum). Controls were performed with PBS or Poly(I:C) + Alum. Dot plot of B220+ cells (PerCP x FSC-A). [file Image_3.tif]

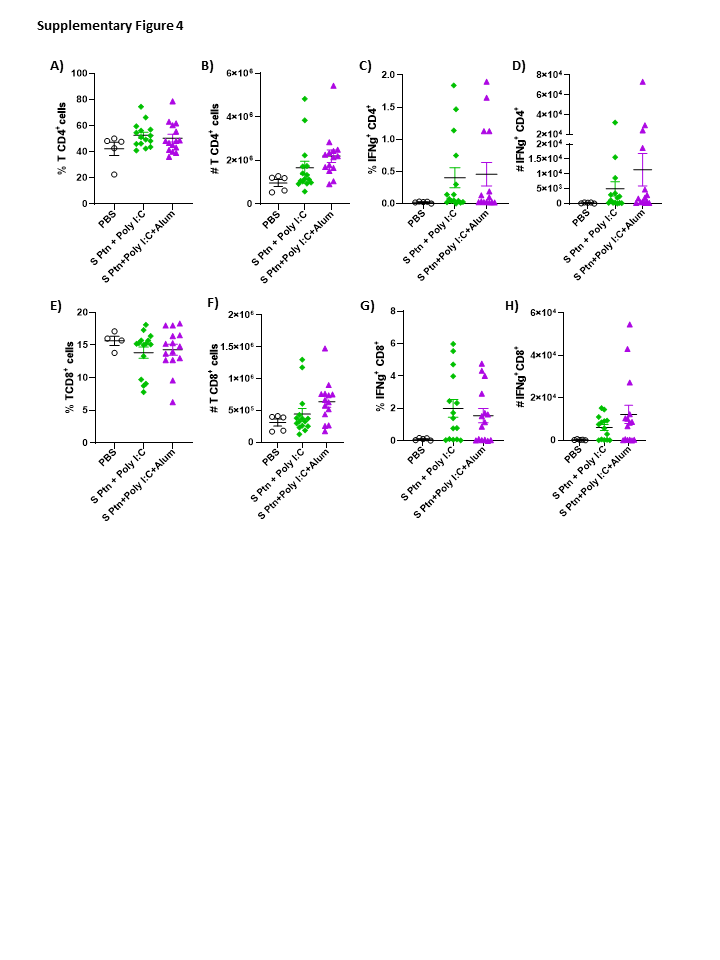

Supplement: Supplementary Figure 4 — Profile of CD4+ and CD8+ T cell response after immunization. Lymphocytes isolated from the draining popliteal lymph node after three immunizations by intradermal immunization with S Ptn plus Poly(I:C) or Poly(I:C) + Alum. Controls were performed with PBS. (A) Percentage of CD4+ T cells. (B) Number of CD4+ T cells. (C) Percentage of IFN-γ+CD4+ T cells. (D) Number of IFN-γ+CD4+ T cells. (E) Percentage of CD8+ T cells. (F) Number of CD8+ T cells. (G) Percentage of IFN-γ+CD8+ T cells. (H) Number of IFN-γ+CD8+ T cells. (SEM; n=5-15). [file Image_4.tif]

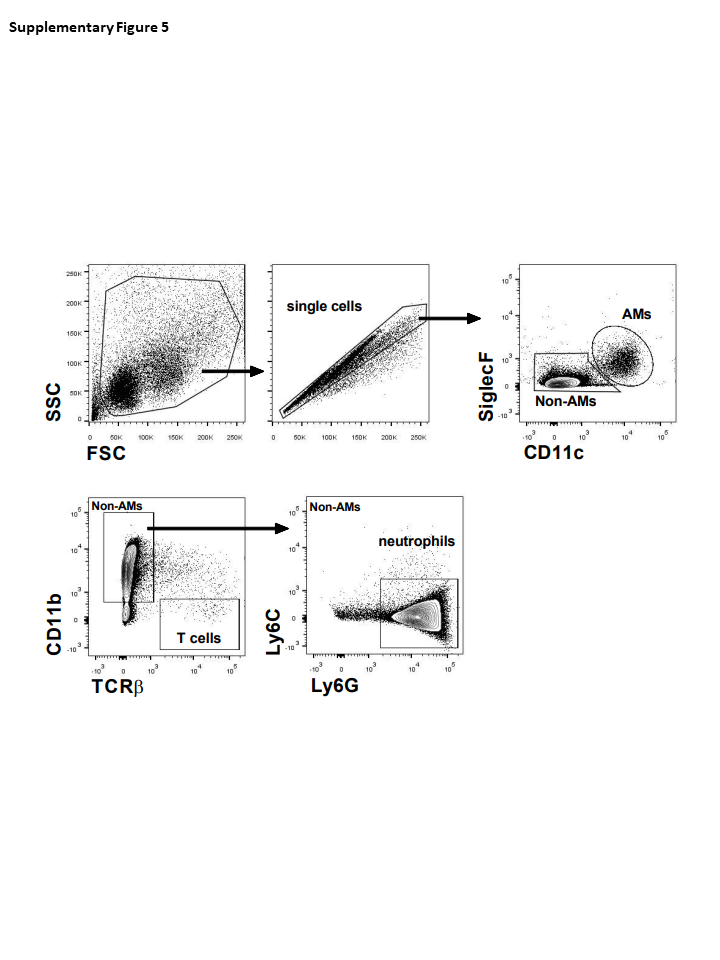

Supplement: Supplementary Figure 5 — Gating strategy for the flow cytometric detection of macrophages, eosinophils, T cells, and neutrophils in BALF. BALF cells were first gated on a forward scatter/side scatter (FSC-A/SSC-A) and then on single cells using FSC-A/FSC-H. A sequential gating strategy was used to identify cellular populations expressing specific markers: alveolar macrophages (AMs) (SiglecF+CD11c+), eosinophils (SiglecF+CD11c-), T cells (CD11b-TCRβ+), and neutrophils (CD11b+Ly6C-Ly6G+). [file Image_5.tif]

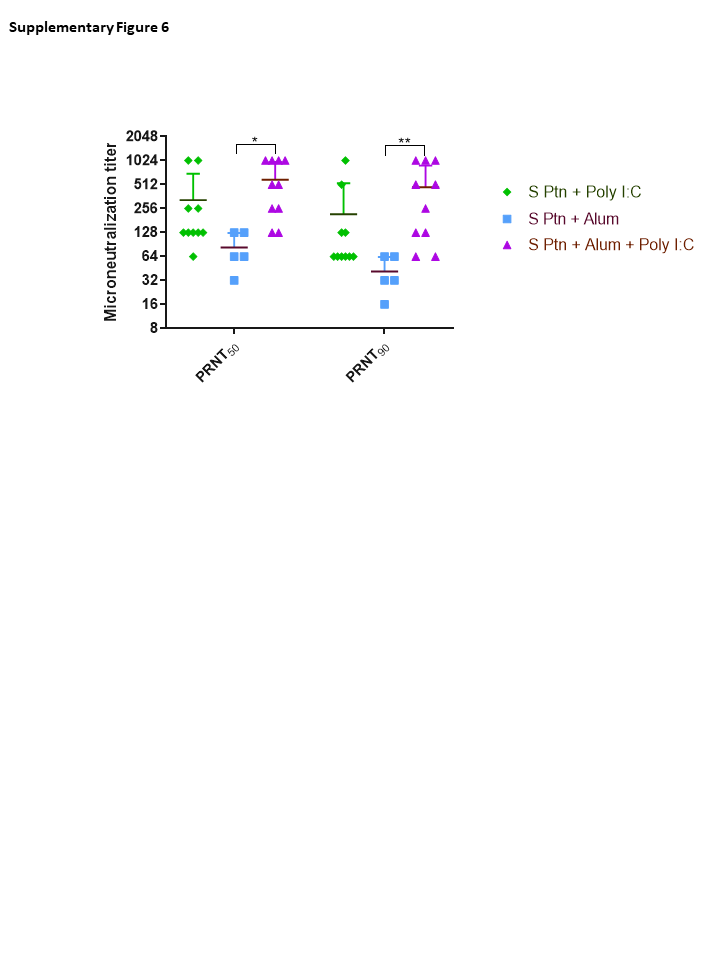

Supplement: Supplementary Figure 6 — Spike protein + Poly(I:C) + Alum and Spike protein + Poly(I:C) induced high titers of neutralizing antibodies against Delta variant. Titers of neutralizing antibodies were determined in vitro by neutralization assay after three immunizations and PRNT50 and PRNT90 for mice plasma collected 7 days after third immunization. Figure representative of 2 experiments and shown as mean ± S.D. and analyzed by one-way ANOVA with Tukey’s post hoc test. *p<0.05 (SD; n=5-10). [file Image_6.tif]

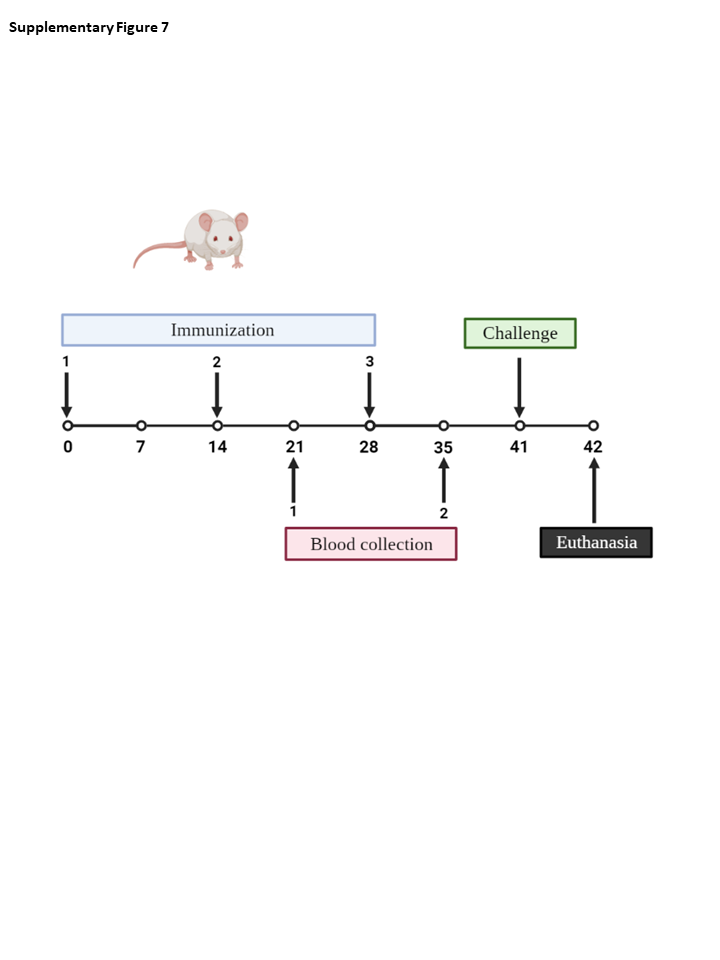

Supplement: Supplementary Figure 7 — Protocol of Immunization. The animals received three immunizations, with the same dosage, with an interval of fourteen days between each dose. Blood samples was collected after two and three immunizations. Then, mice were challenge with inactivated SARS-CoV-2 and 24h later the mice were euthanized. [file Image_7.tif]
